# Functional Head Impulse Test in Professional Athletes: Sport-Specific Normative Values and Implication for Sport-Related Concussion

**DOI:** 10.3389/fneur.2019.00387

**Published:** 2019-04-30

**Authors:** Fausto Romano, Giovanni Bertolini, Daniel Agostino, Dominik Straumann, Stefano Ramat, Nina Feddermann-Demont

**Affiliations:** ^1^Department of Neurology, University of Zurich, Zurich, Switzerland; ^2^Clinical Neuroscience Center, University Hospital of Zürich, Zurich, Switzerland; ^3^Swiss Concussion Center, Zurich, Switzerland; ^4^Department of Computer, Electric and Biomedical Engineering, University of Pavia, Pavia, Italy

**Keywords:** functional head impulse test, VOR (Vestibulo-Ocular Reflex), sport related concussion, visual acuity (VA), professional athletes, acceleration, normative data, vestibulogram

## Abstract

Dizziness, slow visual tracking, or blurred vision following active head (or body) movements are among the most common symptoms reported following sport-related concussion, often related to concurrent dysfunctions of the vestibular system. In some cases, symptoms persist even if bedside and auxiliary standard vestibular tests are unremarkable. New functional tests have been developed in recent years to objectify neurological alterations that are not captured by standard tests. The functional head impulse test (fHIT) requires the patient to recognize an optotype that is briefly flashed during head rotations with various angular accelerations (2,001–6,000 deg/s^2^) and assesses the proportion if correct answers (*pca*). 268 active professional athletes (23.70 ± 5.32y) from six different sports were tested using fHIT. *Pca* were analyzed both pooling head acceleration in the range of 2,001–6,000 deg/s^2^ and computing a single *pca* value for each 1,000 deg/s^2^ bin in the range 2,001–8,000 deg/s^2^. No significant difference (*p* = 0.159) was found between responses to head impulses in the plane of horizontal (*pca*: 0.977) and vertical semicircular canals (*pca*: 0.97). The sport practiced had a major effect on the outcome of the fHIT. Handball players achieved a better performance (*p* < 0.001) than the whole athlete group, irrespective of the direction of head impulses. The *pca* achieved by athletes practicing snowboard, bob and skeleton were instead significantly below those of the whole athlete group (*p* < 0.001) but only when vertical head impulses were tested. Overall, *pca* declined with increasing head acceleration. The decline was particularly evident in the range not included in the standard fHIT exam, i.e., 6,001–8,000 deg/s^2^ for horizontal and 5,001–8,000 deg/s^2^ for vertical head impulses. When vertical head impulses were tested, athletes practicing snowboard, bob and skeleton (non-ball sports) showed, beside the lower overall *pca*, also a steeper decline as a function of vertical head acceleration. The findings suggest that: (1) functional VOR testing can help understanding sport-specific VOR requirements; (2) the fHIT is able to detect and objectify subtle, sport-specific changes of functional VOR performance; (3) if sport-specific normative values are used, the fHIT test procedure needs to be optimized, starting from the highest acceleration to minimize the number of head impulses.

## Introduction

Sport-related concussion (SRC, syn; mild traumatic brain injury), the most frequent form of traumatic brain injury, is a clinical diagnosis (1, 2) frequently based on the results of symptom scales and neurological, neuropsychological and balance examinations. Accurate assessment following a head impact is challenging. Since different domains may be affected, no clinical test, or biomarker can currently make the diagnosis in isolation (1). Furthermore, although concussion typically results in the rapid onset of short-lived functional impairments that resolve spontaneously, clinical recovery might be prolonged and unpredictable in selected cases (2, 3). Symptoms may remain, change or newly evolve during the following days, months, or years (2, 4) and continue even after alterations of clinically observable parameters have normalized. Consequently, if examinations are performed several days after the impact, the reported symptoms might not match with the picture emerging from the results of objective clinical tests (5). The latter situation, unfortunately, is not uncommon in professional athletes, where a short period of rest followed by rapid return to routine training and match play is often attempted and referral to specialized centers occurs only when symptoms fail to subside spontaneously.

These considerations call for the implementation of functional tests that, integrating the currently valid clinical tests, allow objective assessment of the functional impairment causing the symptoms occurring in real life conditions and, when considering athletes, during professional sport activities.

Dizziness has a prominent role among the symptoms reported following concussion with an incidence between 35 and 80% in athletes [it is the second most common symptom following headache in SRC (1, 6)] and up to 80% in the general population of concussed patients (7). Even more important, the presence of dizziness immediately after the impact is the single greatest risk factor for longer symptoms remission time and delayed recovery (8–10), with 18–20% of the patients still symptomatic after 2–5 years (11, 12). The high occurrence of dizziness following a head impact clearly suggests that impairments along the pathways processing visual and/or vestibular signals are common in concussed patients (13, 14). The most frequent peripheral cause of dizziness and vertigo after concussion is benign paroxysmal positional vertigo (BPPV). It is caused by the mechanical effect of the impact, dislodging calcium carbonate concrements from the otolith organs. The concrements, once loose, may enter the semicircular canals and, as a result, perturb the normal flow of endolymph required to sense head motion (15–17). Dizziness following concussion, however, may also be consequence of other peripheral, i.e., labyrinthine damage (18), or central, i.e., brainstem and cerebellar lesions (17, 19), vestibular impairment. Extensive assessment of the vestibular function is therefore critical to identify the cause of dizziness following concussion (5).

The rotational vestibulo-ocular reflex (VOR) aims at stabilizing vision by generating eye movements precisely compensating for head rotation. Testing the VOR is an excellent method to test the functioning of the vestibular organs, since a direct, rapid three-neurons pathway connects the semicircular canals with the eye muscles (20). The head impulse test (and its video-oculography based adaptation, the video head impulse test—vHIT) (21–23) quantifies the VOR responses to head accelerations at frequencies (1–5 Hz) (24) in the upper range of natural head movements (25). It consists in asking the patient to keep fixation on a stationary target while the examiner imposes a small, abrupt rotation of his/her head along the plane of a single canal pair. Randomly alternating impulses in both directions, the test assesses the functioning of each single canal. Since each pair of semicircular canals works in in a push-pull mechanism, a head impulse in one direction inhibits the afferents from one canal and excites those from the other. Accordingly, if the impulse exceeds a velocity threshold (around 200 deg/s) (26), the afferents of the inhibited side reach inhibitory cut off (i.e., zero firing rate) and the response to the amount of head velocity above threshold is accounted for by the excited canal only. The test outcome (denoted as “VOR gain”) is the ratio of a measure of eye movement to the corresponding head movement (e.g., eye velocity/head velocity) averaged within a time window. The VOR gain objectively quantifies the percentage of head movement compensated by the ocular motor response. From a clinical perspective, the assessment of VOR gain with vHIT is therefore of primary importance to exclude that dizziness in a concussed patient derives from an impairment of the semicircular canals.

On the other hand, while VOR gain tells us whether the compensatory eye movements indicate a normal functioning of the semicircular canals, it does not directly assess the functional effectiveness of such movement, i.e., if gaze stabilization was sufficient to permit clear vision. It is indeed not uncommon that athletes who suffered a SRC report blurred vision or fogginess during fast head movements, even in presence of a normal VOR gain (27). This may originate from different impairments ranging from suboptimal visual processing (slower visual processing speed or reduced retinal slip tolerance) (28, 29), to an insufficiently long period of optimal visual stability during the head impulse. In fact, visual stabilization is achieved by a complex combinations of eye movements consisting of an optimal tradeoff between head position and head velocity compensation (30) at any instant of the head movement. Testing the functional effectiveness of VOR for head impulses is therefore complementary, not identical, to the vHIT.

Currently, two tests have been developed to assess the functional performance of the VOR during passive head impulses: the dynamic visual acuity test (DVA) (31–33) and the functional head impulse test (fHIT) (34–36). They both assess VOR function by requiring the patient to identify an optotype briefly presented during the passive head impulses. Their outcome, however, is profoundly different. The DVA first determines the minimum size optotype that can be recognized while keeping the head still (static visual acuity, measured in term of the visual angle it subtends—logMAR) and then quantify the decrease in visual acuity occurring during head impulses (called dynamic visual acuity) keeping the range of head angular accelerations and speeds as consistent as possible (37, 38). The fHIT, after assessing the static visual acuity, quantifies the percentage of correctly recognized optotypes using a relatively large, fixed-size optotype (0.6 logMAR larger than the static visual acuity) during head impulses scanning a wide range of head angular accelerations (2,001–6,000 deg/s^2^). The two systems provide therefore two different assessments. The DVA, by measuring the decrease of a functional parameter, quantifies how much head movements with high acceleration and high frequency degrade visual acuity, but it does not evaluate if and how this degradation leads to a practical impairment in daily activities. The fHIT, by measuring performance in a task that should be flawlessly executed by healthy individuals [the 0.6 logMAR increase was selected to minimize error in healthy individuals in the range 1,001–4,000 deg/s^2^ (39)], identifies how much the actual stabilization ability is impaired as head acceleration increases, but does not provide a measure of the actual degradation of visual acuity (40). In short, the DVA quantifies the amount of lost visual acuity during head motion while the fHIT the residual performance in a standardized, simple visual task.

The current paper focuses on the fHIT, since it directly assesses VOR functional performance across different head accelerations. We hypothesize that the fHIT may indeed capture the specific performance level required for the professional activity of athletes and help therefore to objectify the impairment underlying dizziness and blurred vision occurring on the field. Accordingly, since diagnosis with functional vestibular tests is based on the comparison of the patients' behavior with that of healthy individuals, we speculate that group-specific references are necessary for professional athletes.

The aim of the current study was therefore to evaluate the outcomes of the fHIT in a large population of healthy professional athletes, investigate differences among sports with high risks of concussion and quantify the effect of different head accelerations. To our knowledge, since no previous study investigated how the fHIT (or even DVA) outcomes vary as a function of the head acceleration, this study is also the first to address this question on the functional testing of the vestibular system.

## Materials and Methods

**Figure 1:**
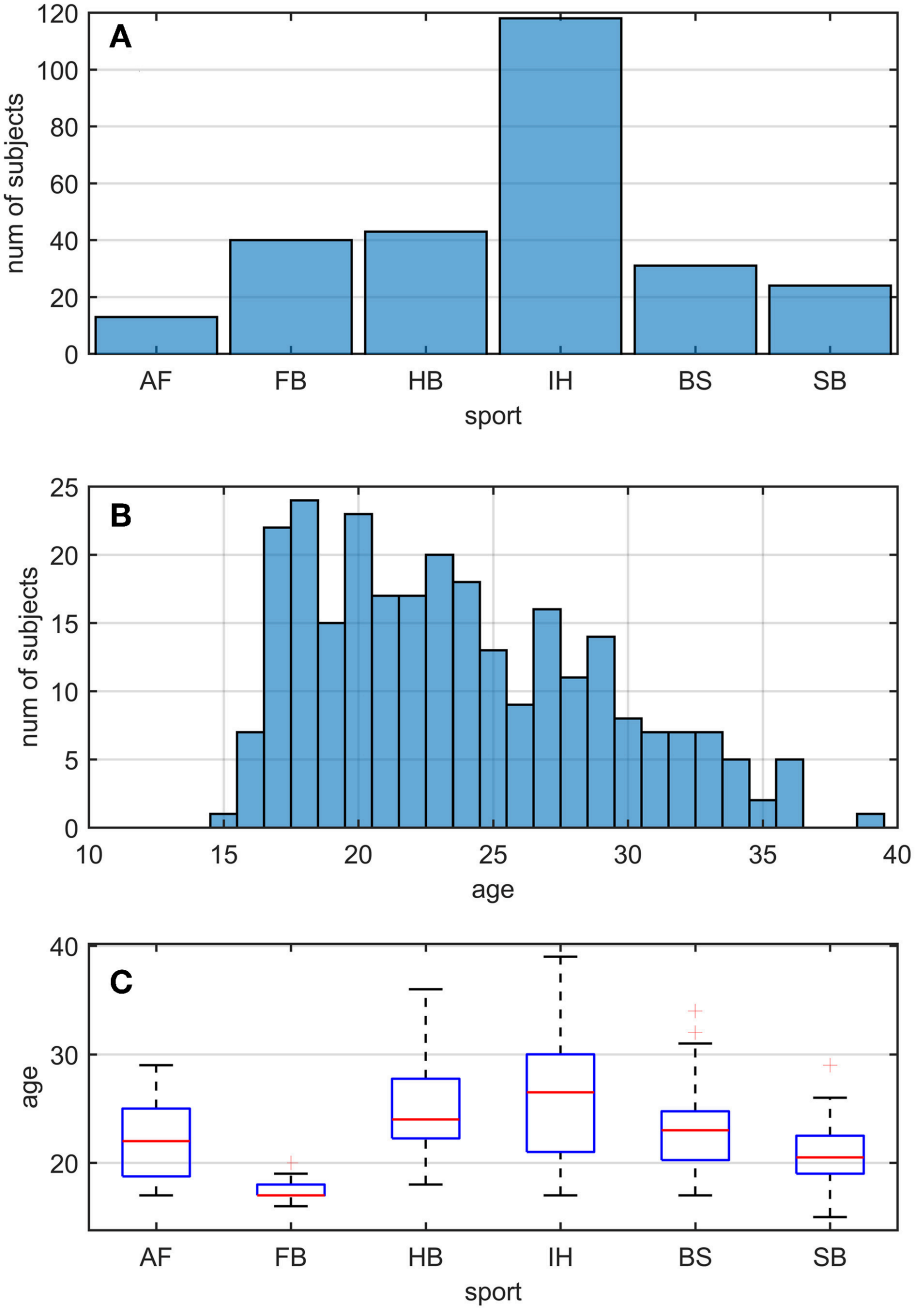
Sport and age distributions of athletes. AF, American Football; FB, Football; HB, Handball; IH, Ice Hockey; BS, Bob and Skeleton; SB, Snowboard. **(A)** Number of athletes divided by sport. **(B)** Age distribution in the athletes. As visible, for each bin of one y.o. (expect for 15, 35, and 39), our data counted at least five athletes. **(C)** Boxplots of age distribution per sports. Despite sport groups did not cover overlapping age ranges, no relationship between and test performance was found (see Pooled Bin Approach: Between Sports Analysis in Results).

Two hundred and sixty nine active athletes (23.70 ± 5.32 [15, 39] y.o.; average ± sd [min, max]) were included (named *whole athlete group*). They were considered professional in six different sports considered at risk of concussion [four contact and ball-based sports (American football, football, handball, ice hockey) and two non-contact winter sports (bob and skeleton, snowboard) (41)—see Table 1 and Figures 1A,C for additional details]. The athletes' ages were adequately distributed across the range tested, counting at least 5 athletes per year (Figure 1B).

**Table 1:** Sport and age distributions of athletes.

**Sport type**		**Number of subjects**	**Gender distribution**		**Age (mean ± std y.o.)**
			**M**	**F**	
Ball sports	American Football (AF)	13	13	0	21.8 ± 3.7
	Football (FB)	40	40	0	17.5 ± 1.2
	Handball (HB)	43	43	0	25.1 ± 4.0
	Ice Hockey (IH)	118	118	0	26.2 ± 5.3
Non-ball sports	Bob and Skeleton (BS)	31	21	10	23.5 ± 4.2
	Snowboard (SB)	24	13	11	20.8 ± 3.1

As control, 26 healthy individuals (27.26 ± 6.19 [20, 40] y.o.) not practicing any sport activities at professional level were included (named *control group*). Data were extracted from the normative population provided by the company commercializing the fHIT device that was used in this study (Beon Solution, Zero Branco (TV), Italy), discarding the subjects above 40 y.o.

The study protocol was approved by the local ethics committee (cantonal ethics commission Zürich, KEK-ZH-2018-01168) and was in accordance with the ethical standards laid down in the 2013 Declaration of Helsinki for research involving human subjects. Written informed consent was obtained from each participant. For the participants under the age of 18, written informed consent was obtained from the parents or guardians of participants.

### Experimental Setup and Procedure

The experimental setup and the testing procedure replicated the one first introduced by (35), validated on patients with vestibular deficits (40, 42), and now commercially distributed as fHIT (Beon Solutions, Italy).

In brief, all recordings were obtained with the athlete seated on a chair placed at 150 cm distance from a computer screen connected with the fHIT device. The athletes using mean of static visual acuity correction (e.g., contact lenses), were requested to wear them during the test. A trained operator performed subsequent head impulses, consisting of brief, small rotatory movements impressed with both hands to the head of the athlete in the plane of each semicircular canals pair. An optotype, the Landolt ring, was displayed for 80 ms on the computer screen (60 Hz) when the imposed head angular acceleration and velocity exceeded pre-defined thresholds. The athlete was requested to recognize the ring orientation reporting it on a keypad showing all possible ring orientations. The Landolt ring allows 8 different orientations reducing the probability of random correct answers respect to the Sneller E optotype, which typically is presented only in 4 orientations (43). The ring size was adjusted according to a preliminary test of static visual acuity, increasing the smallest line seen by 0.6 LogMAR (39). No time limit was set to provide the answer after each impulse. To test the vertical semicircular canals, the chair was rotated 45 degrees (to the left for the left-anterior-right-posterior semicircular canal plane and to the right for the right-anterior-left-posterior plane). The athlete was asked to counterrotate the head to look straight at the screen and impulses were performed in the sagittal plane of the athlete's body. This procedure allows testing the recognition of the optotype during impulses with the eye starting from the primary position (i.e., the visual axis is aligned with head straight ahead axis), reducing the risk that occlusions from the eyelids or the constraints of the oculomotor system (e.g., Listing law) affect the test outcome. The software of the fHIT device guided the initial positioning of the head to ensure that the impulses are performed in the planes of each pair of vertical canals.

The operator performed a minimum of 10 head impulses in each direction for each semicircular canal plane (Left horizontal [11, 36] [min, max]; Right horizontal [12, 35]; Left Anterior [11, 32]; Right Posterior [11, 31]; Right Anterior [11, 29]; Left Posterior [10, 33]) attempting to achieve accelerations covering all the 1,000 deg/s^2^ bins from 3,000 deg/s^2^ to 8,000 deg/s^2^ according to the classification of the fHIT software (e.g., the 3,000 deg/s^2^ bin includes the acceleration range between 2,001 and 3,000 deg/s^2^).

### Data Analysis

The fHIT software automatically separated the trials (i.e., head impulses) according to the acceleration bins defined above and the semicircular canal stimulated. For each bin of acceleration *a*, two variables were used for the data analysis, the number of trials *t*_*a*_ performed by the tester and the number of correct answers *c*_*a*_ (e.g., for *a* = 5,000 deg/s^2^, *t*_5000_ and *c*_5000_ were used). Data were imported in MATLAB Version R2016b (The Mathworks, Natick, MA, USA) and further data analysis was performed using custom-written programs.

Data from different semicircular canal planes were grouped, creating two “virtual” semicircular canals, a horizontal and a vertical canal. Specifically, for the “virtual” horizontal canal, the *t*_*a*_ and *c*_*a*_ of left and right horizontal semicircular canals were grouped together, while left and right semicircular canals of the anterior and posterior canals were grouped into the *t*_*a*_ and *c*_*a*_ of the “virtual” vertical canal. Such approach was considered since it is not in the scope of the current study to investigate natural asymmetries between and within the left and right vestibular organs and it is reasonable to assume that the overall population of the tested athletes has negligible systematic asymmetries.

### Pooled Bin Approach

#### fHIT Procedure

As established by the fHIT procedure, the test performance was assessed estimating the proportion of correct answer (*pca*). The *pca* was computed using the procedure proposed by (35), called pooled bin approach *(pb)*. Specifically, the data in the acceleration bins ranging from 3,000 to 6,000 deg/s^2^ were pooled together and the *pca* was computed as follow:

(1) $$\begin{array}{l} pca^{pb}=\frac{\sum_{s=1}^{n_{subjs}}\sum_{a=1}^{n_{bins}}c_{sa}}{\sum_{s=1}^{n_{subjs}}\sum_{a=1}^{n_{bins}}t_{sa}} \end{array}$$

with *n*_*bins*_ = 4 (i.e., the number of acceleration bins) and *n*_*subjs*_ = the number of athletes considered for the *pca* estimation. It is worth noting that the *pb* approach does not consider between-subject variability, creating an *pca* estimation of the whole group (Equation 1). The 7,000 and 8,000 deg/s^2^ bins were excluded in the *pb* approach, according to the procedure used by the fHIT software when comparing a single patient to the reference population. To compare the estimated *pca* between different groups, the Zeta-test for two proportions was used, keeping in line with the procedure used by the fHIT software.

#### Comparisons Between PCA of the PB approach

The *pb* approach was used to perform three analyses: between-canals, between-sports, and *whole athlete group* vs. *control group*.

*Between-canals analysis*: The analysis aimed at assessing potential differences between the athletes' performances during horizontal and vertical head movements. The $pca_{hor}^{pb}$ and the $pca_{vert}^{pb}$, obtained using Equation 1 on the data of the virtual horizontal and semicircular canals, respectively, were compared.

*Between-sports analysis*: The analysis aimed to compare the performance of each subgroup of athletes pooled by sport to the *whole athlete group*. Six comparisons were performed, one per sport, testing whether the${\;pca}_{sport}^{pb}$ of the athletes from one sport (e.g., $pca_{HB}^{pb}$ for handball, see Table 1 for sport coding) was different to the $pca_{ath}^{pb}$ of the *whole athlete group*. In this series of comparisons, the α used for the Zeta test was corrected using Bonferroni procedure to reduce the type-1 error due to multiple comparisons $(\alpha_{bonf}=\;\frac{\alpha }{n_{comparison}}=\frac{0.05}{6}=0.0083$; with *n*_*comparison*_ = *n*_*sports*_ = 6). The analysis procedure was performed separately for the horizontal and vertical canal.

*Whole athlete group vs. control group analysis*: The analysis aimed at assessing whether the fHIT discriminates between *whole athlete group's* and *control group's* performances, comparing the $pca_{ath}^{pb}$ of athlete *group* to the one of *control group* (${pca}_{ctr}^{pb}$). Such procedure was performed only for the horizontal canal, as no normative data of anterior and posterior canals was available.

### Single Bin Approach

To test whether a relationship between the proportion of correct answers and head accelerations exists in the *whole athlete group*, a single bin *(sb)* procedure was used. Compared to the *pb* approach (Equation 1), where trails with different head accelerations (up to 6,000 deg/s^2^) were pooled, here the proportion of correct answers was computed separately for each bin of acceleration (*a*) as follows:

(2) $$\begin{array}{l} pca_{a}^{sb}=\frac{\sum_{s=1}^{n_{subjs}}c_{sa}}{\sum_{s=1}^{n_{subjs}}t_{sa}} \end{array}$$

with *n*_*subjs*_ = the number of athletes considered for the *pca* estimation.

#### Relationship Between PCA and Head Acceleration

To avoid postulating any assumption on the relationship between the *pca* and the head acceleration, all 15 possible pairs of the six $pca_{a}^{sb}$ (one per acceleration bins between 3,000 deg/s^2^ and 8,000 deg/s^2^), were compared using the Zeta-test for proportions (i.e., 15 zeta-tests). According to the Bonferroni correction, a *p*-value lower than 0.003 was considered statistically significant ($\alpha_{bonf}=\;\frac{\alpha }{n_{comparison}}=$ 0.05/15). Analyses were performed separately for horizontal and vertical canals.

#### Relationship Between PCA and Head Acceleration Within Sports

The same approach was used to evaluate the effects of acceleration within each sport. To limit the number of statistical comparisons and the associate *p*-value corrections, two subgroups of athletes were defined using one characteristic of sports, namely if a ball is used or not. The two groups were named the ball and non-ball group, respectively. The ball group included American football, soccer, handball and ice-hockey athletes, while the non-ball group included snowboard, bob and skeleton athletes. As described for the *whole athlete group*, six $pca_{a}^{sb}$ were estimated (one for each acceleration bin) and compared each pair of accelerations (15 comparisons). Additionally, comparison between ball and non-ball groups was performed for each bin of acceleration (e.g., $pca_{a=3000\deg/s^{2}}^{sb\;-Ball}\;vs\;pca_{a=3000\deg/s^{2}}^{sb-\;nonBall}$), adding 6 more comparisons. Bonferroni procedure was used to correct the significance level of z-tests ($\alpha_{bonf}=\;\frac{\alpha }{n_{comparison}}=$ 0.05/21 = 0.0024).

## Results

### Pooled Bin Approach

#### Horizontal vs. Vertical Canals

**Figure 2:**
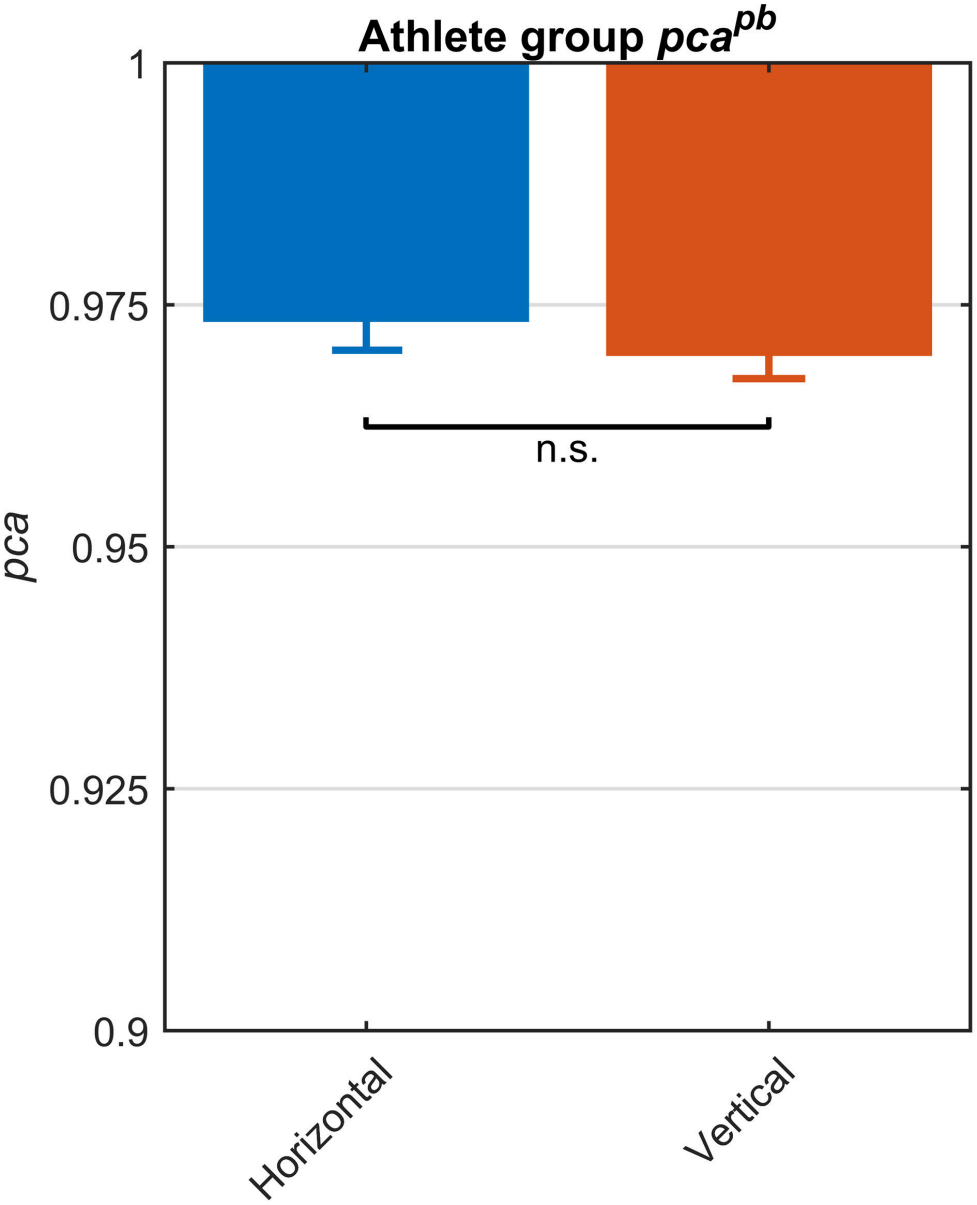
Proportions of correct answers (*pca*) for each vestibular canal in the *whole athlete group*. *Pca* of impulses performed in the horizontal (blue) and vertical canals (red) were computed using the pooled approach (Equation 1) considering only head movements in the range of 2,001–6,000 deg/s^2^. Zeta distribution was used to estimate the lower bound of 95% confidence interval for *pca*, as the number of trials (i.e., performed head movements) was large enough (>>200). As visible, the fHIT performance was not significant influenced by canals (n.s.).

The fHIT test outcome computed over the *whole athlete group* according to the standard procedure of the fHIT software (i.e., pooling the acceleration bins in the range 3,000–6,000 deg/s^2^) evidenced no differences between head impulses in the plane of the horizontal and of the vertical canals (Figure 2). The *pca* of vertical canals ($pca_{vert}^{pb}$ = 0.970) was only ~0.3% less than the *pca* of the horizontal canals ($pca_{hor}^{pb}$ = 0.972); accordingly, the Zeta-test did not show a significant difference (*p* = 0.159).

#### Between Sports Analysis

**Figure 3:**
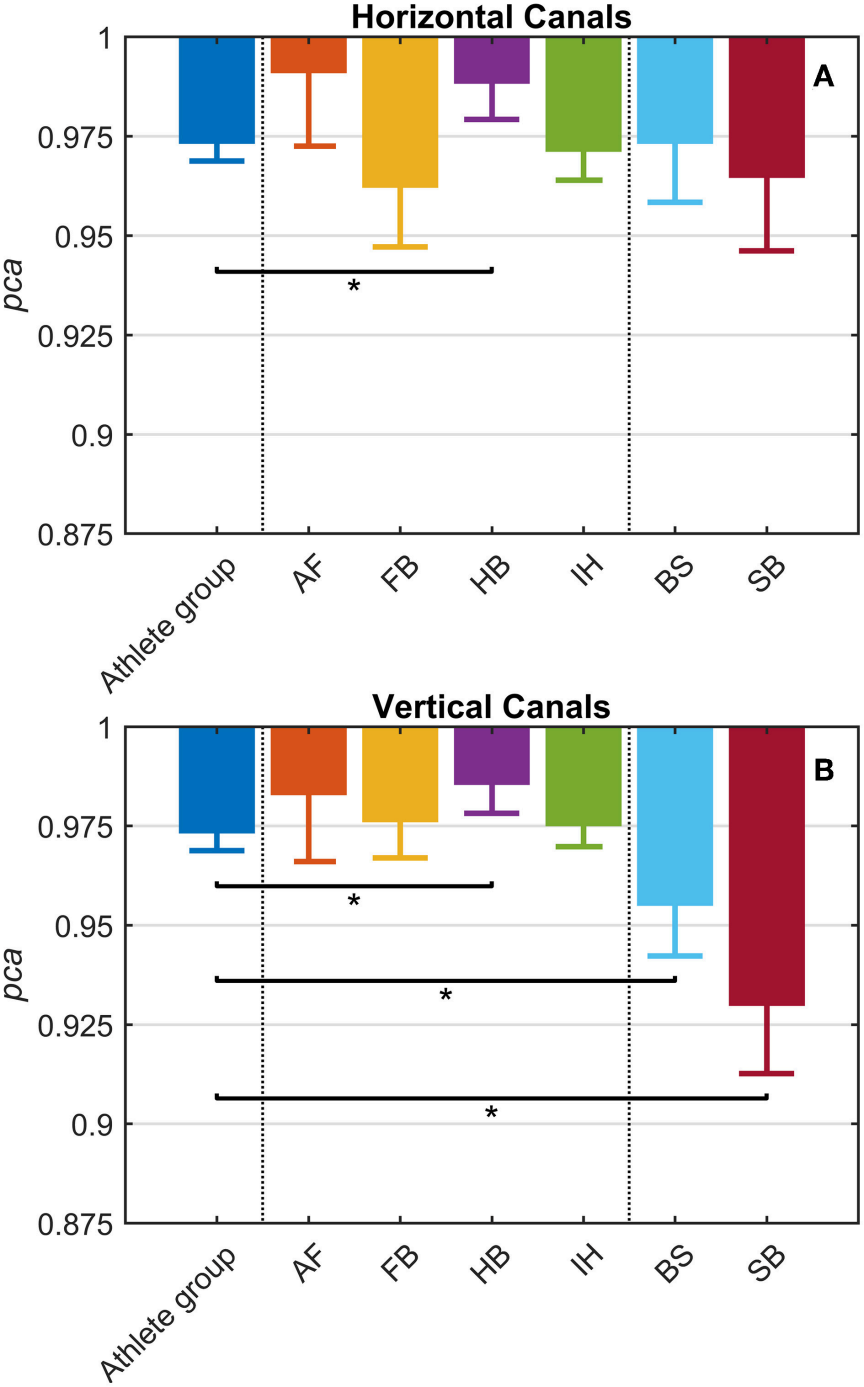
Proportion of correct answers (*pca*) in the *whole athlete group* and single sport. AF, American Football; FB, Football; HB, Handball; IH, Ice Hockey; BS, Bob and Skeleton; SB, Snowboard. For ease of visualization, the dashed vertical lines separate the *whole athlete group* from the ball sports (first vertical line) and the non-ball sport (second vertical line). All *pca* were computed using the pooled approach (Equation 1) considering only head movements in the range of 2,001–6,000 deg/s^2^. Zeta distribution was used to estimate the lower bound of 95% confidence interval (*c.i*.) for *pca*, as the number of trials (i.e., head movements) was large enough (>>200). The asterisk (^*^) represents the statistically significant comparisons (*p*-value < α_*bonf*_ = 0.0083). **(A)** *Horizontal canals*. HB players (violet bar) showed a significant higher fHIT performance (*pca* >0.975) respect to the *whole athlete group* (dark blue bar). **(B)** *Vertical canals*. The fHIT performance in vertical canal was clearly lower *pca* in the non-ball sport players (i.e., BS and SB, respectively the light blue and dark red bar) respect to the *whole athlete group*. HB player, instead, confirmed the same finding for the horizontal canals, achieving a higher *pca* for impulses performed in the vertical canals as well.

Each sport showed distinctive fHIT performance levels for head impulses in the plane of the horizontal and vertical canals, suggesting that the specific sports practiced by an athlete has a relation with his/her *pca* (Figure 3). Such observation is also confirmed by the multiple comparisons (see Table 2). Despite a non-significant difference was found between head impulses in the plane of the horizontal and vertical canals in the *whole athlete group*, between-sport analysis revealed that a canal–sport interaction was present. The sport-specific $pca_{sport}^{pb}$ estimated on the tested athletes are presented in Table 2.

**Table 2A:** Single sport vs. *whole athlete group*—horizontal canals.

**Compared sport**		**${pca}_{sport}^{pb}$**	**${pca}_{athletes}^{pb}$**	***P*-value**
Ball sports	AF	0.991	0.973	0.043
	FB	0.962	0.973	0.045
	HB	0.989	0.973	0.002
	IH	0.971	0.973	0.595
Non-ball sports	BS	0.973	0.973	0.991
	SB	0.965	0.973	0.187

*PCA was estimated using the pooled bin approach (Equation 1). Using the Bonferroni correction, only p < 0.0083 (highlighted in gray) were consider statistically significant*.

Only handball players (HB) showed significantly higher performance than the *whole athlete group* irrespective of the tested canal (horizontal canals: *p* = 0.002; vertical canals: *p* < 0.001). The *pca* of HB players was ~1.5% higher than the *pca* of the *whole athlete group* for both the horizontal canals ($pca_{HB}^{pb}=$ 0.989 vs. $pca_{ath}^{pb}=$0.973) and the vertical canals ($pca_{HB}^{pb}=$ 0.986 vs. $pca_{ath}^{pb}\;=$ 0.970).

Two out of three winter sports, snowboard (SB) and bob and skeleton (BS), showed a *pca* lower than the *pca* of the *whole athlete group*, but limited to head impulses stimulating the vertical canals (BS: *p* = 0.002; SB: *p* < 0.001—light blue and dark red bars of Figure 3B). Despite the *pca* of the SB athletes appears to be lower than the one of the *whole athlete group* also for impulses in the plane of the horizontal canals (Figure 3A and Table 2A), the difference was not significant. The non-uniform distribution of age and gender among sports (Figure 1 and Table 1) poses the question of whether these factors rather than the sport may influence the fHIT outcome.

**Figure 4:**
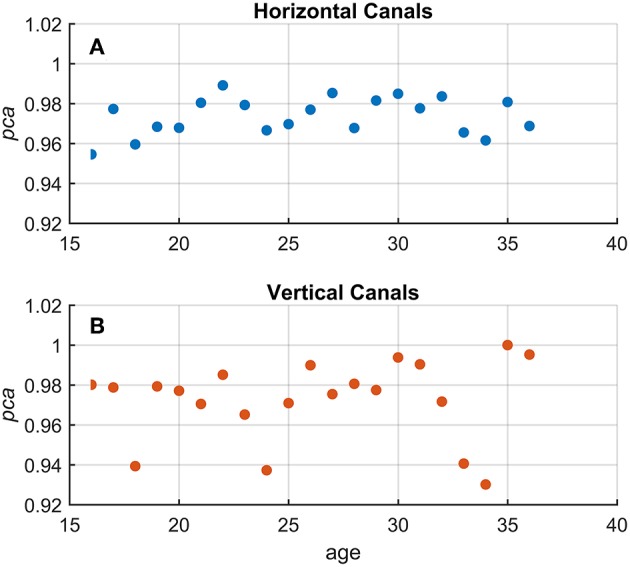
Relationship between age and proportion of correct answers (*pca*) in the *whole athlete group*. Each point in the scatter plot represents a *pca* estimated in subgroups of athletes pooled by age (1-year-old bin). All *pca* were computed using the pooled approach (Equation 1) considering only head movements in the range of 2,001–6,000 deg/s^2^. As evident in the two scatter plots for fHIT performed in the horizontal (**A** and blue points) and vertical (**B** and red points), the point dispersal did not argument any potential correlation and/or interaction between the age and *pca* (i.e., test performance). The values of *pca* appear, indeed, randomly distributed regardless of age in a range between 1–0.95 and 1–0.92 for the horizontal and vertical canals, respectively.

Using Equation 1, a *pca* was estimated grouping the athletes by age, using one-year-old bins. No significant association between age and *pca* was found (Figure 4) both for head impulses in the plane of the horizontal (Kendal's τ coefficient = 0.13; *p* = 0.420) and vertical canals (Kendal's τ coefficient = 0.143; *p* = 0.386).

**Figure 5:**
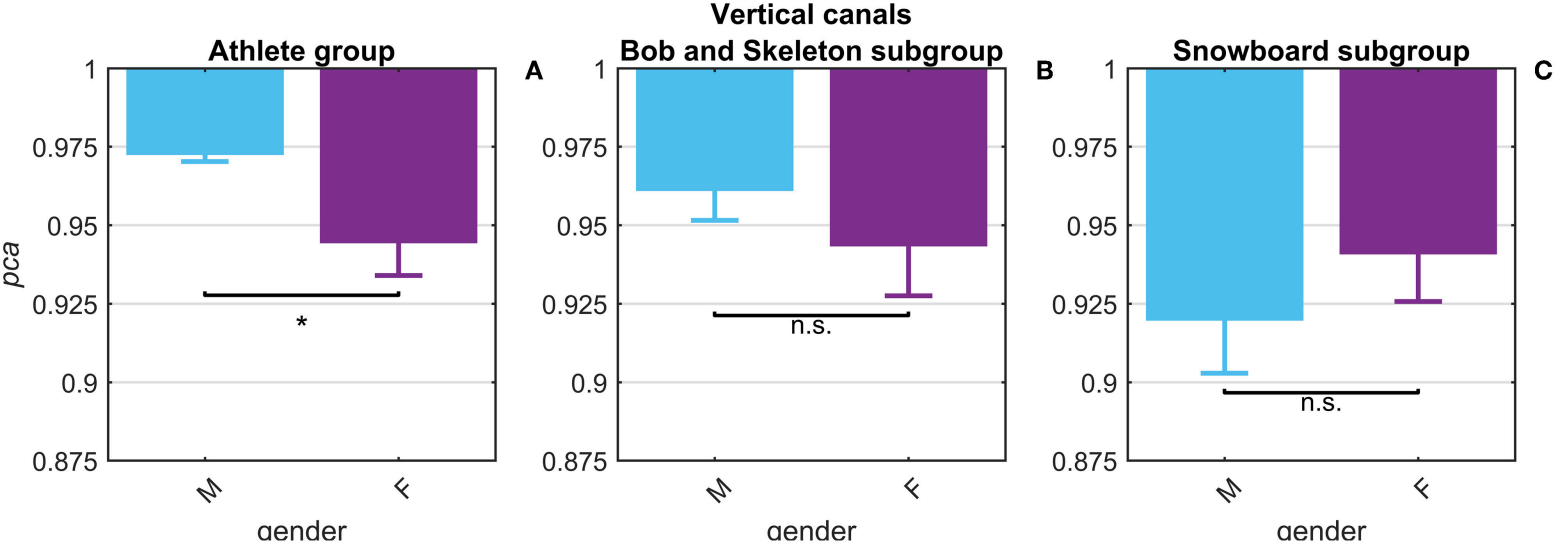
Gender effect on proportion of correct answers (*pca*) for impulses in the plane of the vertical canals. Each bar represents the complement of *pca (*i.e*., 1-pca)* estimated in subgroups of athletes pooled by gender (female: violet bars; male: light blue bar). All *pca* were computed using the pooled approach (Equation 1) considering only head movements in the range of 2,001–6,000 deg/s^2^. Confidence intervals for *pca* were estimated as in Figure 3. The asterisk (^*^) represents the statistically significant comparisons (*p*-value < α = 0.05). Using the *athlete group* **(A)**, the *pca* estimated in the subgroups of female athlete was significant lower than the *pca* of male athlete. The effect of gender on fHIT performance, however, was not present considering also the sport factor **(B,C)**. The significant difference between genders, indeed, was induced by a non-uniform distribution of female athletes that were recruited only in the two sports (BS and SB) with the lowest *pca* for impulses performed in the vertical canals (see Table 2A).

The potential gender effect was tested comparing the *pca* estimated for the female and male athletes. No significant difference (*p* = 0.14) was found in fHIT performance for head impulses in the plane of the horizontal canals ($pca_{female}^{pb}=$ 0.965 vs. $pca_{male}^{pb}=$ 0.974). The results of the head impulses in the plane of the vertical canals evidenced that male athletes had a significantly higher *pca* (*p* < 0.001) than female ones ($pca_{female}^{pb}=$ 0.945 vs. $pca_{male}^{pb}=$ 0.973, see Figure 5A). The sample of female athletes, however, was composed entirely by athletes of the two sports with the lowest performance for head impulses in the plane of the vertical canals (i.e., BS and SB, Table 2B). The comparison of the *pca* of the male and female athletes subgroups within these two sports (Figures 5B,C), showed no significant difference between female and male athletes both in BS ($pca_{female}^{pb}=$ 0.944 vs. $pca_{male}^{pb}=$ 0.961, *p* = 0.104), and in SB ($pca_{female}^{pb}=$ 0.941 vs. $pca_{male}^{pb}=0.919$, *p* = 0.133). Furthermore, since even the non-significant differences between genders have opposite signs in the two sports (i.e., female are better in SB and worse in BS), it is therefore evident that the finding of an overall gender difference is due to the non-uniform distribution of genders between sports (Table 1—i.e., all female athletes in the sample are from sports with lower fHIT outcomes).

**Table 2B:** Single sport vs. *whole athlete group*—vertical canals.

**Compared sport**		**${pca}_{sport}^{pb}$**	**${pca}_{athletes}^{pb}$**	***P*-value**
Ball sports	AF	0.983	0.970	0.077
	FB	0.976	0.970	0.127
	HB	0.986	0.970	<0.001
	IH	0.975	0.970	0.052
Non-ball sports	BS	0.955	0.970	0.002
	SB	0.930	0.970	<0.001

*PCA was estimated using the pooled bin approach (Equation 1). Using the Bonferroni correction, only p < 0.0083 (highlighted in gray) were consider statistically significant*.

#### Athlete Group vs. Control Group

To investigate whether sport activity at professional level affects athletes' performance in fHIT, their *pca* was compared to the one of the *control group*, extracted by the normative dataset of the fHIT.

Despite the two groups were not age-matched (*p* = 0.001), their age ranges were comparable and, as confirmed in Figure 4, no relationship between age and *pca* was found in the range 16−39 y.o.

**Figure 6:**
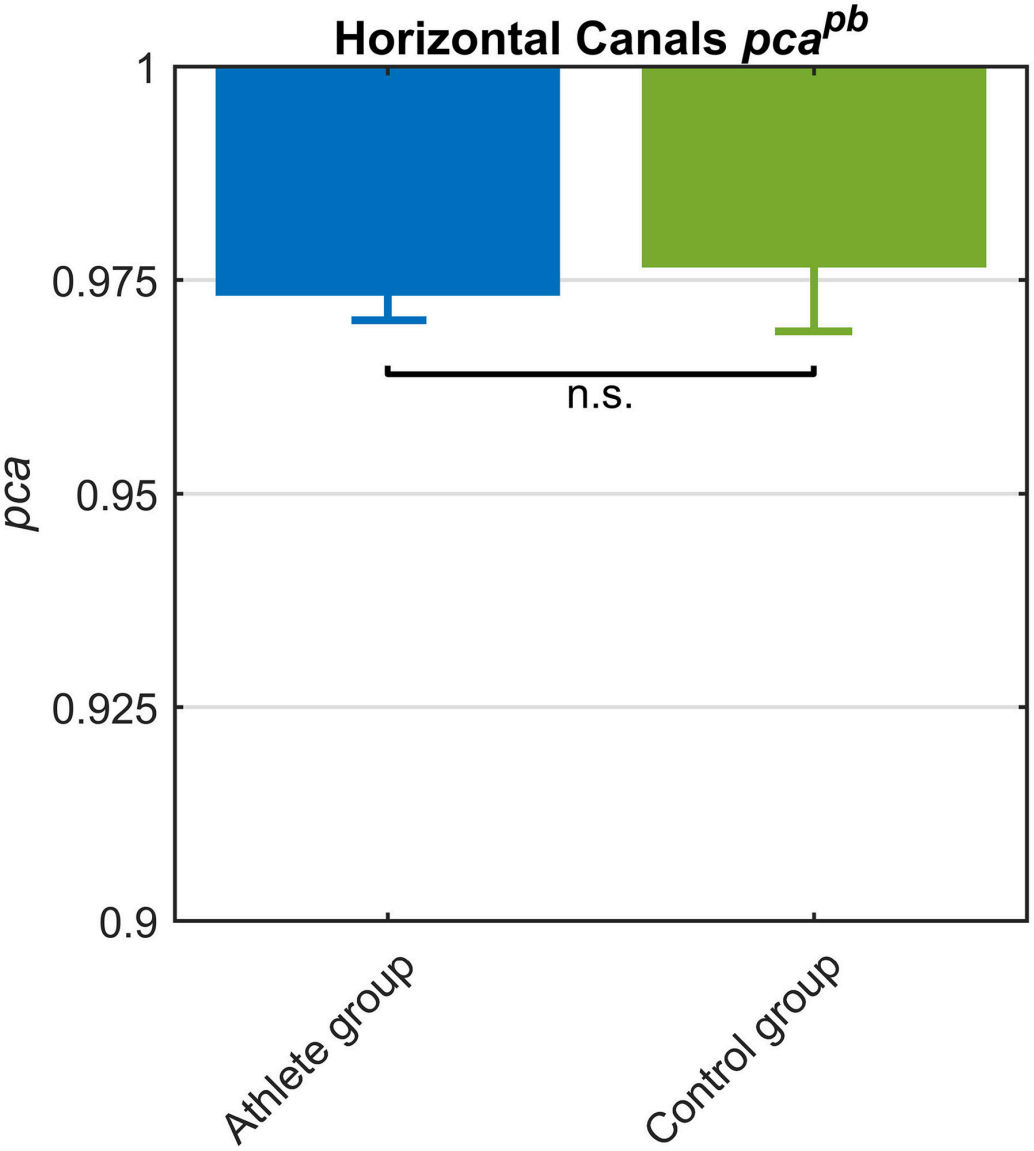
Proportion of correct answers (*pca*) in *whole athlete group* (blue) and *control group* (green). The two *pca* for the impulses performed in the plane of the horizontal canals were computed using the pooled approach (Equation 1) considering only head movements in the range of 2,001–6,000 deg/s^2^. Confidence intervals for *pca* were estimated as in Figure 3. The *whole athlete group* and *control group* showed a comparable performance for impulses performed in the horizontal canals, as showed by the overlapping *c.i.*. Accordingly, the *pca* were revealed no significant (n.s.) different by Zeta test (see section Results).

The *athletes group* and the *control group* showed comparable *pca*^*pb*^ (Figure 6) and a clear overlap between the lower limits of 95% confidence intervals. Such tendency was confirmed by Zeta-test. Accordingly, the test did not show a significant difference (*p* = 0.089) between the *pca* of *whole athlete group* ($pca_{ath}^{pb}=$ 0.973) and *control group* ($pca_{ctr}^{pb}\;=$ 0.977).

Since the *pca* of the HB players was significantly higher than the *one* of the *whole athlete group* (Table 2A), it was worth it to compare HB subgroup to *control group*. The *pca* of HB players revealed ($pca_{HB}^{pb}=$ 0.989) a significantly higher performance (*p* = 0.034) than *control group* ($pca_{ctr}^{pb}=$ 0.977).

### Single Bin Approach

#### Relationship Between Head Acceleration and PCA in the Athlete Group

**Figure 7:**
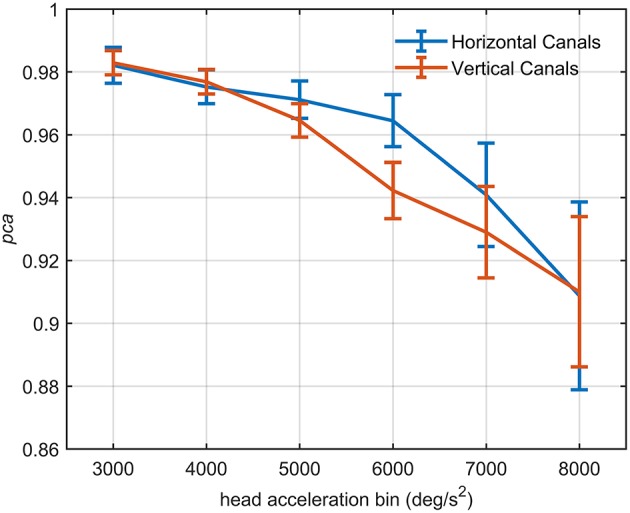
Proportion of correct answers (*pca*) of the *whole athlete group* at different head acceleration bins. The error bars represent the *pca* and 95% confidence intervals estimated for bin of acceleration (3,000–8,000 deg/s^2^)*. Pca* were computed using the single approach (Equation 1) for impulses performed in the plane of the horizontal (blue line) and vertical (red line) canals. Confidence intervals for *pca* were estimated as in Figure 3. The relationship between head acceleration and test performance appeared different between the two tested vestibular canals. The vertical canals showed, indeed, a significant decrease of the *pca* already from the 5,000 deg/s^2^ bin, while for the horizontal canal, the worsening in performance occur only after the 6,000 deg/s^2^ bin (Table 3).

The influence of head acceleration on the fHIT outcome is visible in Figure 7, where the athletes' *pca* clearly decreases for the highest head accelerations tested. The effect distinctively emerges by the series of comparisons between the pca of all possible pairs of acceleration bins (Tables 3A,B).

**Table 3A:** *Whole athlete group*: *p*-values of the multiple comparisons of *pca* between head acceleration bin—horizontal canals.

**Acceleration bin**	***pca***	**3000 deg/s^**2**^**	**4000 deg/s^**2**^**	**5000 deg/s^**2**^**	**6000 deg/s^**2**^**	**7000 deg/s^**2**^**	**8000 deg/s^**2**^**
		0.982	0.975	0.971	0.965	0.941	0.909
3000 deg/s^2^	0.982	-	0.206	0.048	0.005	<0.001	<0.001
4000 deg/s^2^	0.975	0.206	-	0.454	0.076	<0.001	<0.001
5000 deg/s^2^	0.971	0.048	0.454	-	0.314	0.001	<0.001
6000 deg/s^2^	0.965	0.005	0.076	0.314	-	0.028	<0.001
7000 deg/s^2^	0.941	<0.001	<0.001	0.001	0.028	-	0.129
8000 deg/s^2^	0.909	<0.001	<0.001	<0.001	<0.001	0.129	-

*PCA was estimated using the single bin approach (Equation 2). Using the Bonferroni correction, only p <0.0042 (highlighted in gray) were consider statistically significant*.

**Table 3B:** *Whole athlete group*: *p*-values of the multiple comparisons of *pca* between head acceleration bin—vertical canals.

**Acceleration bin**	***pca***	**3000 deg/s^**2**^**	**4000 deg/s^**2**^**	**5000 deg/s^**2**^**	**6000 deg/s^**2**^**	**7000 deg/s^**2**^**	**8000 deg/s^**2**^**
		0.983	0.977	0.965	0.942	0.929	0.910
3000 deg/s^2^	0.983	-	0.090	<0.001	<0.001	<0.001	<0.001
4000 deg/s^2^	0.977	0.090	-	0.023	<0.001	<0.001	<0.001
5000 deg/s^2^	0.965	<0.001	0.023	-	<0.001	<0.001	<0.001
6000 deg/s^2^	0.942	<0.001	<0.001	<0.001	-	0.216	0.024
7000 deg/s^2^	0.929	<0.001	<0.001	<0.001	0.216	-	0.295
8000 deg/s^2^	0.910	<0.001	<0.001	<0.001	0.024	0.295	-

*PCA was estimated using the single bin approach (Equation 2). Using the Bonferroni correction, only p < 0.0042 (highlighted in gray) were consider statistically significant*.

For impulses in the plane of the horizontal canals, a significant worsening of fHIT performance was shown for the range of acceleration 6,001–8,000 deg/s^2^ compared to the one between 2,001 and 5,000 deg/s^2^ (see Table 3A for *p*-values). Specifically, the *pca* of the 7,000 deg/s^2^ bin ($pca_{athletes}^{7000\deg/s^{2}}=$ 0.941) was significantly higher than the pca of the 3000 ($pca_{athletes}^{3000\deg/s^{2}}=$ 0.982, *p* < 0.001), 4000 ($pca_{athletes}^{4000\deg/s^{2}}=$ 0.975, *p* < 0.001) and 5,000 deg/s^2^ ($pca_{athletes}^{5000\deg/s^{2}}=$ 0.971, *p* < 0.001) bins, showing a decrease of ~4.1, ~3.5, and ~3%, respectively. As expected, such decreases remain significant (Table 3A) for higher acceleration as well (i.e., 8,000 deg/s^2^ bin, $pca_{athletes}^{8000\deg/s^{2}}=$ 0.909).

An earlier decline of performance with increasing head acceleration is observed for impulses in the planes of the vertical canals (Figure 7, red curve). The worsening of fHIT outcome was indeed already significant for accelerations 1,000 deg/s^2^ lower than for impulses in the plane of the horizontal canals. Specifically, the Zeta tests revealed a significant decrease (*p* < 0.001) of *pca* in the range 4,001–8,000 deg/s^2^ compared to the range 2,001–4,000 deg/ s^2^ (Table 3B). This worsening is particularly evident in the two bins not considered in the pooled bin approach (7,000 and 8,000 deg/s^2^), where the performance decreases exceed 5% ($pca_{athletes}^{7000\deg/s^{2}}=$ 0.929, $pca_{athletes}^{8000\deg/s^{2}}=$ 0.910) of the *pca* achieved in the lowest accelerations bins (i.e., $pca_{athletes}^{2000\deg/s^{2}}=$ 0.983, $pca_{athletes}^{3000\deg/s^{2}}=$ 0.977).

#### Relationship Between Head Acceleration and PCA in the Ball and Non-ball Subgroups

**Figure 8:**
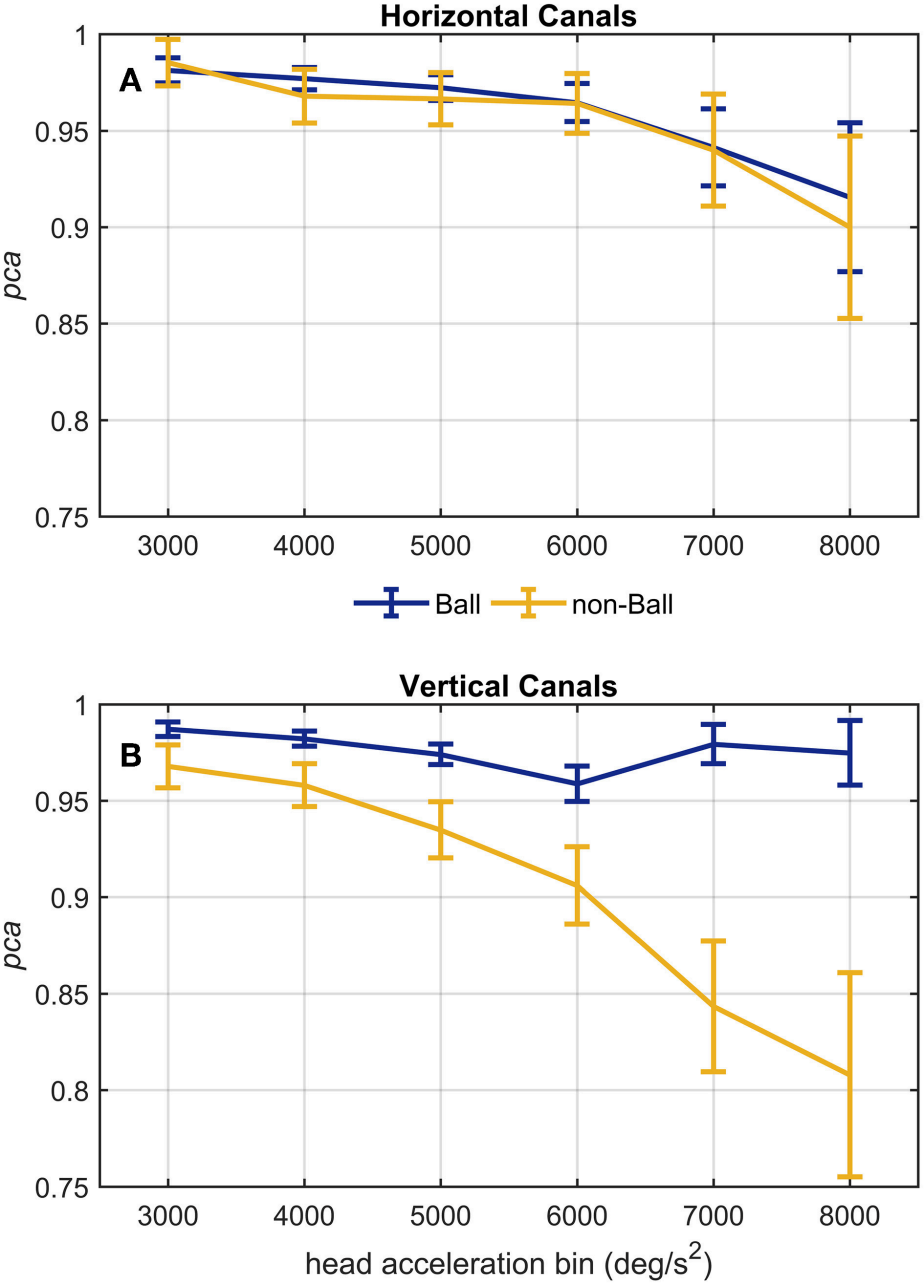
Proportion of correct answers (*pca*) of ball and non-ball groups at different head acceleration bins. The error bars represent the *pca* and 95% confidence intervals estimated for bin of acceleration (3,000–8,000 deg/s^2^)*. Pca* were computed using the single approach (Equation 1) for impulses performed in the plane ot the horizontal **(A)** and vertical **(B)** canals. Confidence intervals for *pca* were estimated as in Figure 3. Dark blue and yellow lines represent the *pca* estimated for the ball and non-ball groups, respectively. **(A)** The relationship between head acceleration and test performance appeared comparable between the two subgroups of athletes (ball and non-balls) for the impulses collected in the plane of the horizontal canals. No significant difference was, indeed, found for every acceleration bin (Table 4A). **(B)** As visible, the two ball and non-ball subgroups showed two peculiar relationships of head acceleration and *pca*. The comparisons between the subgroups showed a significant higher *pca* of the ball group than the non-ball group, irrespective of head acceleration bins (Table 4B).

A further analysis of the relationship between head acceleration and *pca* was performed on two subgroups of athletes, separating the sports where no balls are used (BS and SB) from those where the athletes needs to focus their attention on a ball-like object (AF, FB, HB, IH). The performance for head impulses in the plane of the horizontal canals did not differ between the two groups, as evidenced by the two overlapping curves in Figure 8A. The zeta tests did not show significant differences when comparing the *pca* of ball and non-ball groups within each bin of acceleration tested (*p* > 0.36, for individual *p*-values see Table 4A).

**Table 4A:** Ball vs. non-ball sports: comparisons of *pca* at different head acceleration bin—horizontal canals.

	**Head acceleration bin**					
**Sport type**	**3000 deg/s^**2**^**	**4000 deg/s^**2**^**	**5000 deg/s^**2**^**	**6000 deg/s^**2**^**	**7000 deg/s^**2**^**	**8000 deg/s^**2**^**
Ball	0.981	0.977	0.972	0.9645	0.941	0.915
Non-ball	0.985	0.968	0.967	0.964	0.940	0.900
*p*-value	0.851	0.358	0.608	0.900	0.902	0.839

*PCA was estimated using the single bin approach (Equation 2). Using the Bonferroni correction, only p < 0.0027 (highlighted in gray) were consider statistically significant*.

A different finding emerged from the analysis of the head impulses in the plane of the vertical canals (Figure 8B). The *pca* of the group of athletes from ball sports was significantly greater than the one of non-ball sports, regardless of the head accelerations (*p* < 0.006, for the single p-values see Table 4B). Pooling together all bins (from 3,000 up to 8,000 deg/s^2^), the non-ball group showed a ~5% lower *pca* than the ball group ($pca_{non-ball}^{3000-8000\deg/s^{2}}=$ 0.929, $pca_{ball}^{3000-8000\deg/s^{2}}=$ 0.978).

**Table 4B:** Ball vs. non-ball sports: comparisons of *pca* at different head acceleration bin—vertical canals.

	**Head acceleration bin**					
**Sport type**	**3000 deg/s^**2**^**	**4000 deg/s^**2**^**	**5000 deg/s^**2**^**	**6000 deg/s^**2**^**	**7000 deg/s^**2**^**	**8000 deg/s^**2**^**
Ball	0.987	0.982	0.974	0.959	0.979	0.975
Non-Ball	0.968	0.958	0.935	0.906	0.843	0.808
*p*-value	0.001	<0.001	<0.001	<0.001	<0.001	<0.001

*PCA was estimated using the single bin approach (Equation 2). Using the Bonferroni correction, only p < 0.0027 (highlighted in gray) were consider statistically significant*.

Despite the difference in the fHIT outcome emerges at all head accelerations, the two curves in Figure 8B evidence that the two subgroups present two different relationships between head acceleration and *pca*. In the non-ball group, the *pca* of the 3,000 and 4,000 deg/s^2^ bins was significantly higher than that of the last three bins (6,000, 7,000, and 8,000 deg/s^2^ - *p*-values in Table 5B). Furthermore, the *pca* decrease did not “slowdown” at higher accelerations. The *pca* in 5,000 deg/s^2^ bin ($pca_{non-ball}^{5000\deg/s^{2}}=$ 0.939) was still significantly higher than the ones in the 7,000 and 8,000 deg/s^2^ bins ($pca_{non-ball}^{7000\deg/s^{2}}=$ 0.843, $pca_{non-ball}^{8000\deg/s^{2}}=$ 0.807), and the *pca* in 6,000 deg/s^2^ bins was also significantly higher than the one in the 8,000 deg/s^2^ bin ($pca_{non-ball}^{6000\deg/s^{2}}=$ 0.906).

In the ball group, only the comparisons involving the two lowest acceleration bins (3,000 and 4,000 deg/s^2^, *p*-values in Table 5A) were significant. Specifically, the *pca* in the 3,000 deg/s^2^ bin ($pca_{non-ball}^{3000\deg/s^{2}}=$ 0.987) was significantly higher only than the *pca* in the 5,000 and 6,000 deg/s^2^ bins ($pca_{non-ball}^{5000\deg/s^{2}}=$ 0.974, *p* = 0.002; $pca_{non-ball}^{6000\deg/s^{2}}=$ 0.969, *p* < 0.001), while more surprisingly only in the 6,000 deg/s^2^ bin the ball group showed a significant reduction (*p* < 0.001) of the *pca* compared to the 4,000 deg/s^2^ ($pca_{non-ball}^{4000\deg/s^{2}}=$ 0.974). These isolated differences suggest that the decrease of reading performance with increasing acceleration in the ball group, if any, is relatively weak and is masked by the increase of measure variability observed at higher accelerations.

**Table 5A:** Ball sports: *p*-values of the multiple comparisons of *pca* between head acceleration bin—vertical canals.

**Acceleration bin**	***pca***	**3000 deg/s^**2**^**	**4000 deg/s^**2**^**	**5000 deg/s^**2**^**	**6000 deg/s^**2**^**	**7000 deg/s^**2**^**	**8000 deg/s^**2**^**
		0.987	0.982	0.974	0.959	0.979	0.975
3000 deg/s^2^	0.987	-	0.183	0.002	<0.001	0.250	0.214
4000 deg/s^2^	0.982	0.183	-	0.047	<0.001	0.783	0.571
5000 deg/s^2^	0.974	0.002	0.047	-	0.015	0.577	0.887
6000 deg/s^2^	0.959	<0.001	<0.001	0.015	-	0.043	0.321
7000 deg/s^2^	0.979	0.250	0.783	0.577	0.043	-	0.896
8000 deg/s^2^	0.975	0.214	0.571	0.887	0.321	0.896	-

*PCA was estimated using the single bin approach (Equation 2). Using the Bonferroni correction, only p < 0.0027 (highlighted in gray) were consider statistically significant*.

**Table 5B:** Non-ball sports: *p*-values of the multiple comparisons of *pca* between head acceleration bin—vertical canals.

**Acceleration bin**	***pca***	**3000 deg/s^**2**^**	**4000 deg/s^**2**^**	**5000 deg/s^**2**^**	**6000 deg/s^**2**^**	**7000 deg/s^**2**^**	**8000 deg/s^**2**^**
		0.968	0.958	0.935	0.906	0.843	0.808
3000 deg/s^2^	0.968	-	0.3816	0.006	<0.001	<0.001	<0.001
4000 deg/s^2^	0.958	0.3816	-	0.0454	<0.001	<0.001	<0.001
5000 deg/s^2^	0.935	0.006	0.0454	-	0.0630	<0.001	<0.001
6000 deg/s^2^	0.906	<0.001	<0.001	0.0630	-	0.0075	0.0012
7000 deg/s^2^	0.843	<0.001	<0.001	<0.001	0.0075	-	0.410
8000 deg/s^2^	0.808	<0.001	<0.001	<0.001	0.0012	0.410	-

*pca were estimated using the single bin approach (Equation 2). Using the Bonferroni correction, only p < 0.0027 (highlighted in gray) were consider statistically significant*.

## Discussion

According to the fHIT outcome, functional performance of the vestibular ocular reflex (VOR) was close to perfection in the professional athletes tested, granting them a clear vision during head motion. The reading performance, quantified by an overall proportion of correct answer >97% for head accelerations ranging 2,001–6,000 deg/s^2^, was independent of the semicircular canals tested (i.e., horizontal or vertical). Head acceleration, on the other hand, affected reading performance. The effect, which is prominent for accelerations exceeding 6,000 deg/s^2^ (>5% difference to the 3,000 deg/s^2^ bin), differed between horizontal and vertical head impulses, with the latter showing a significant decline already within the range of accelerations used in the standard fHIT outcome measure (i.e., 2,001–6,000 deg/s^2^).

Within the overall high level of performance, sport specific differences were also observed (Table 2). Handball players performed better in the fHIT than the overall population of athletes tested (*whole athlete group*), irrespectively of the plane of head rotations (i.e., both for vertical and horizontal canals stimulations). Athletes practicing bob, skeleton and snowboard performed worse for head impulses in the vertical planes than the overall population of athletes tested (i.e., for vertical canals only). Although age and gender distributions differed between the groups, the absence of any overall correlation between age and the fHIT outcome, and the absence of a gender effect within the sports where both males and females were tested, suggest that age and gender inhomogeneity played no role in the observed sport-specific differences.

At first sight, sport-related differences as small as few percentage points may appear of little importance in relation to the almost perfect response rate of the *whole athlete group*. These values as well as the effect of head acceleration, however, have considerable relevance for three different aspects: (1) to understand the sport-specific requirement for functional VOR performance; (2) to improve the interpretation of the fHIT and clarify its relevance when testing athletes; (3) to adjust the testing procedure of the fHIT with respect to testing athletes.

### Sport-Specific Requirement for Functional VOR Performance

The sport-specific differences may allow to gain insight in the VOR demand posed by different sports and, consequently, in the importance of accurate vestibular assessment for the athletes in the process of return to sport following sport related concussion (SRC). The results evidence that athletes taking part in ball sports (i.e., American football, football, handball and ice hockey) have higher reading performance during vertical head impulses than those from non-ball sports (i.e., bob, skeleton, and snowboard). Such a clear separation between sport types allows speculation on how the different VOR demands occur. Possibly, athletes of ball sports continuously need to rapidly, though precisely, focus on single objects (e.g., the ball/puck or the movement of other players body parts to foresee their action) while repetitively and rapidly moving the head to explore the field or see the ball. For bob, skeleton and snowboard athletes achieving (or recovering after a concussion) a VOR-based gaze stabilization exceeding the requirement for everyday life may not be necessary, since, although they move rapidly, they do not need to focus small visual cues.

The higher functional performance of the horizontal VOR observed in handball players respect to the *whole athlete group*, including football and ice hockey players, is instead more difficult to interpret. Higher visual acuity in dynamic conditions (i.e., recognized in moving target) has been observed in basketball, water polo, volleyball and baseball players (44–47). It is possible to speculate that, for ball-based games with rapid gameplay (e.g., basketball, handball, ice-hockey), the demand to the VOR-based gaze stabilization relates inversely to the size of the field (in small fields targets as ball or other players are closer, thus requiring larger, and thus faster, head motions). Handball, having a rapid gameplay and a smaller field than soccer and hockey may therefore require a higher VOR functional performance. Handball and American football are also the only ball sports tested where the ball is played evenly above and below eye level (but handball has also a smaller field and a faster gameplay), possibly requiring higher performance in the vertical VOR. Such speculation, however, would need confirmation from analysis of head kinematics during the activities of the different sports. The underlying assumption of these speculations is that the hypothesized higher VOR demand of some sports is associated with observing fHIT results higher than normal in the athletes practicing such sport. Accordingly, handball players performed better than the *control group* (data available only for head impulses in the horizontal plane). No difference, however, was observed between the *whole athlete group* and *control group*. This suggests that the level of functional performance of the VOR (as assessed by the fHIT) does not solely dependent from the athletic level of the tested person, but it may be higher in athletes of specific sports.

### Interpretation of the fHIT Results and Its Relevance in Professional Athletes

#### Standard fHIT Outcome (Proportion of Correct Answers for Head Accelerations 2,001–6,000 deg/s^2^)

The fHIT outcome did not differ between the *whole athlete group* and the *control group* (note that data from only 26 controls in a comparable age range were available). This finding may question the clinical relevance of the observed sport-related differences and the suitability of the fHIT in the evaluation of subtle functional VOR impairments that may affect the professional activity of athletes following sport related concussion.

The absence of differences between a wide group of professional athletes (a*thlete group*) and the *control group* is not surprising. The fHIT settings (39) (e.g., size of the presented optotype, duration of the stimulus, etc.) were specifically optimized to null the number of errors committed by normal healthy individuals during head impulses in the accelerations range 1,001–4,000 deg/s^2^. Accordingly, a typical healthy individual is expected to perform very close to 100% correct answers in standard fHIT test (the standard fHIT outcome pools head impulses with accelerations in the range 2,001–6,000 deg/s^2^). While this strategy maximizes specificity (i.e., proportion of healthy individual that are correctly identified by fHIT) with respect to vestibular impairments (35, 40), it pushes the scores of all healthy individuals close to the 100%, reducing the differences between subgroups. The high significance of the observed sport-specific differences suggests however that, when sport-specific performances are present, the fHIT is able to distinguish them (e.g., handball). This makes the fHIT a valid candidate for an objective assessment of the subtle impairments affecting athlete's return to play (2, 48).

Comparing the *whole athlete group* with a small *control group* of undefined healthy non-athletes is not informative, as the compared subgroups are not sufficiently characterized to grant that any difference is actually present. It cannot be excluded that few individuals of the *control group* have unexpected characteristics [e.g., amateur players of sport requiring elevated eye-head coordination such as table tennis (49)] that pose a demand to the VOR similar or even superior to that of some professional sport activities. As discussed above, the level of VOR functional performance does not solely depend on the athletic level of the tested person. Athletes do not necessarily have a superior functional VOR performance, since not every sport, even if it requires rapid movements, is associated with an elevated functional VOR demand.

To better interpret the fHIT results it is important to understand the origin of the observed errors. The sport-specific differences suggest that, if the number of head impulse is high enough, even small numbers of errors cannot be considered lapses, but actual VOR errors (lapses rate is unlikely to generate such an elegant picture of sport-specific differences). The occurrence of errors during head accelerations ranging 2,001–6,000 deg/s^2^ suggests that failure of visual stabilization, although rare, occurs in healthy individuals. This is not surprising. With the video head impulse test (vHIT) VOR gains as low as 0.8 are not considered pathological as they are found in healthy individuals that do not report functional impairments in everyday life [vHIT is often performed up to a 3,000–4,000 deg/s^2^ (24, 50), while acceleration up to 10,000 deg/s^2^ normally occurs during natural locomotion (25)]. An elegant study (30) calculating point-by-point VOR errors (both in position and in velocity) during the whole course of head impulses demonstrated that healthy individuals show errors as big as 10–20% of the desired compensation of the head movement with head accelerations below 10,000 deg/s^2^. During such head impulses, however, only occasional, small corrective saccades are present (30). The absence of relevant corrections suggests that the VOR errors do not systematically affect visual function in real life (no compensatory mechanism had evolved to correct them). The high proportion of correct answers interleaved by occasional errors not attributable to lapses that are found in the fHIT results of the *control group* implies that the VOR errors only occasionally affect the fHIT task. This suggests that the fHIT task is correctly tuned to be a proxy to a VOR challenge comparable to everyday life. In this context, the ability of the fHIT to differentiate significantly higher performances in specific sports further confirms that using the sport specific values, the fHIT can capture the extra functional tuning aimed at minimizing the effect of VOR errors on sport activities requiring elevated visual stability.

In summary, it is possible to speculate that a test outcome below the proportion of correct answers of the normal population (or of the *whole athlete group*) would be related to a functional VOR deficit that may be relevant in everyday life. An outcome lower than a sport-specific normative value only, would instead be related to a functional VOR impairments that affect the specific activities of that sport. According to this interpretation, for sports where no difference with the performance of *control group* is observed, the time course of VOR functional recovery for return to sport activity and return to everyday life should match. A final confirmation of this interpretation, however, requires evidence from testing patients during their return to activity.

### fHIT Vestibulogram (Proportion of Correct Answers as Function of Head Acceleration)

Differently from the vHIT and DVA test, where head velocities must be consistent between head impulses, the fHIT openly requires the examiner to test various head accelerations. This procedure generates a functional vestibulogram, presenting the proportion of correct answers as function of the peak acceleration in the head impulses, divided in 1,000 deg/s^2^ wide bins up to 7,000 deg/s^2^. This unique feature is not yet exploited by the standard outcome metric, which pools the results from all impulses in the range 2,001–6,000 deg/s^2^. Accordingly, no specific restriction on the minimum number of impulses per bin is specified and the functional vestibulogram is provided for visual evaluation only.

The proportion of correct answers of the *whole athlete group* decreased non-linearly as the head acceleration increased (the evaluation was extended to include head impulses up to 8,000 deg/s^2^ to further confirm the trend). For the head impulses in the plane of the horizontal canals this decrease became statistically significant for the 7,000 deg/s^2^ and 8,000 deg/s^2^ bins with respect to the bins ≤ 5,000 deg/s^2^ and ≤ 6,000 deg/s^2^, respectively. This suggests that around 6,000 deg/s^2^ there is a “critical” acceleration above which the reading performance declines with a slope causing significant difference every 2,000 deg/s^2^. For the vertical canals the decline occurs with head accelerations at least 1,000 deg/s^2^ lower (between 4,001 deg/s^2^ and 5,000 deg/s^2^). The slope in the functional vestibulogram, however, also depends on the sport. When vertical semicircular canals are tested, the athletes of non-ball sports had not only an overall lower level of proportion of correct answers (as already evident from the standard fHIT outcome—Table 2B and Figure 3B), but also a faster decline with increasing acceleration (Figure 8B).

Although VOR gain was shown to decline with the velocity of head impulses (51), to our knowledge, this is the first work providing a detailed description of the decline of functional VOR performance with head acceleration, demonstrating that it differs between the planes of head impulses and between different subgroups of healthy individuals. A previous work observed that DVA loss was lower for impulses faster than 100 deg/s head velocity than if only head velocities higher than 150 deg/s were considered (32). A study using the gaze stabilization test (GST—recognition of an optotype repeatedly presented with a fixed size during active head motion) showed a general increase of the visual acuity loss with head velocity testing the range 60–220 deg/s in step of 40 deg/s (52). GST, however, is based on active head movements tested with progressively increasing velocity. Since it is well-known that DVA testing leads to lower visual acuity loss if performed with active than with passive head motion (32), the decline observed with GST may also depends on the additional functions involved with the active testing (e.g., anticipation).

Altogether, the results of the current study provide evidence that the functional vestibulogram has both theoretical and clinical relevance. The decline of VOR functional performance observed with the fHIT test at acceleration within the range of normal head movements suggest that, even in healthy individuals, occasional blurring can occur when the VOR is challenged with intense activities like those required by professional sports. Whether the differences observed among different sports is a consequence of training (i.e., it can also be rehabilitated) or if it is a natural feature differentiating athletes who succeed within one specific sport from those who don't, cannot be inferred based on the current data. The practical implication of the observed trend, however, is that, when assessing professional athletes, testing higher accelerations may be significantly more informative and may shorten the test duration significantly.

### Sport-Specific Normative Values and Testing Procedure for the fHIT

The sport-specific proportion of correct answer identified in the current study allows defining normative values for each sport (Table 6). Although the differences may appear negligible, they may have relevance for the assessment of athletes reporting difficulties in returning to the professional activity. In line with the consideration of the previous paragraph, handball players may require to focus on the ball hundreds of times during the rapid actions occurring during the game and a difference of 1% may impact more than a few of the most critical actions (e.g., scoring) in a single game.

**Table 6A:**
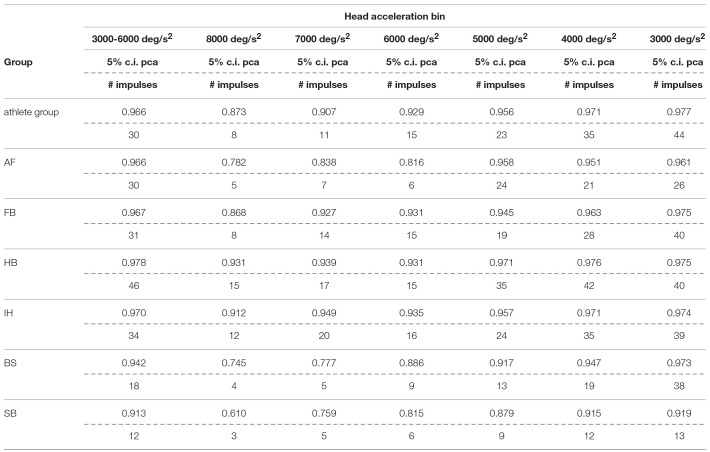
Sport specific lower boundary of *pca* and minimum number of head impulses to identify a deficit—horizontal canals.

*Pca^pb^ were estimated using the pooled bin approach (Equation 1). Pca^sb^ were estimated using the single bin approach (Equation 2). The coefficient intervals were estimated using the Fisher–Snedecor distribution (53), although conservative, a higher reliability of estimate was ensured when in a bin < 200 number of impulses were collected*.

**Table 6B:**
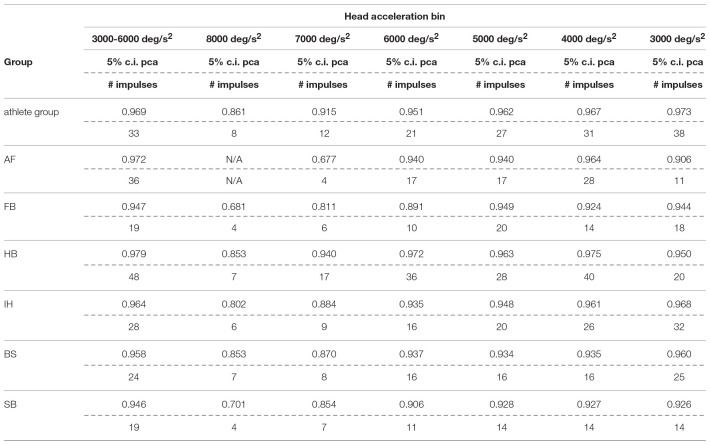
Sport specific lower boundary of *pca* and minimum number of head impulses to identify a deficit—vertical canals.

*Pca^pb^ were estimated using the pooled bin approach (Equation 1). Pca^sb^ were estimated using the single bin approach (Equation 2). The coefficient intervals were estimated using the Fisher–Snedecor distribution (53), although conservative, a higher reliability of estimate was ensured when in a bin < 200 number of impulses were collected*.

If sport-specific normative values were considered, sport-specific testing procedures needs to be defined accordingly. The minimum number of head impulses required to verify whether an athlete achieves its sport-specific level (e.g., to assess sport-specific recovery or sport-specific functional impairment) depends on the distance between the sport-specific proportion of correct answer and 100%. As all the proportions of correct answers are close to 100%, the sport-specific minimum number of required head impulses varies significantly. For example, for the horizontal semicircular canals, handball players (lower bound of correct answer = 97.9%) should be tested with at least 48 impulses. For soccer players (lower bound = 94.7%) the required minimum is only 19 impulses. Table 6 lists the sport-specific, minimum numbers of head impulses required to discriminate the sport-specific lower bound of correct answer. Considering the number of impulses needed for testing the athletes of sports reaching the highest performance, the fHIT may become unpractical (to test all six semicircular canals, 280 head impulses would be the minimum required for a handball player). The observed decline of reading performance with increasing head acceleration, however, may be used to simplify testing. Recalling that during head impulses in the plain of one canal pair, the higher is the acceleration the lower is the contribution of the inhibited semicircular canal (only the excited semicircular canals account for the part of acceleration exceeding the inhibitory cut of the inhibited semicircular canal), a deficit in reading performance observable during head impulses at the lowest accelerations should be more evident during testing at the highest accelerations. Accordingly, an optimal testing strategy would be to start testing the highest acceleration bin and, only if a deficit is present, progressively decrease the acceleration to identify where the pathological behavior stops. With such strategy, a handball player would require a minimum of 17 head impulses at 7,000 deg/s^2^ (the maximum acceleration currently displayed on the fHIT interface) per semicircular canal to be identified as healthy, i.e., comparable to the number of impulses used in a valid head impulse test (51, 54, 55).

In conclusion, the results of the current study suggest that fHIT can be used to characterize the functional vestibular performance of athletes and establish sport-specific reference values. For the sports associated with higher scores, the sport specific-reference values can possibly help to differentiate between a functional VOR performance sufficient for ordinary activities of daily living and one for sport-related activities at professional level only. Furthermore, as the *whole athlete group* showed a sport-specific decrease of VOR functional performance, the results suggest performing the fHIT starting with head impulses at higher accelerations (6,000–8,000 deg/s^2^). This strategy reduces the number of impulse necessary to identify a deficit. All together the fHIT demonstrated a sport-specific sensitivity that supports further extensive tests, focusing on athletes who suffered SRC, to verify sensitivity and specificity for this patients' population. Direct evidence from patients during their return to sport will also be required to confirm that the fHIT can help to identify subtle functional impairments that may become relevant in the athletes when challenged by their professional activity.

## Ethics Statement

The study protocol was approved by the local ethics committee (cantonal ethics commission Zürich, KEK-ZH-2018-01168) and was in accordance with the ethical standards laid down in the 2013 Declaration of Helsinki for research involving human subjects. Informed consent was obtained from each subject.

## Author Contributions

FR supported the implementation of the research study, supported the data acquisition, analyzed the data, interpreted the results, and wrote the manuscript. GB supported the conceivement and the implementation of the research study, acquired the data, interpreted the results and wrote the manuscript. DA supported the management of the research study, acquired the data, participated in the interpretation of the results and critically revised the manuscript. DS participated in the interpretation of the results and critically revised the manuscript. SR supported the data analysis, participated in the interpretation of the results and critically revised the manuscript. NF-D conceived and implemented the research study, managed the research study and the data acquisition, participated in the interpretation of the results and critically revised the manuscript.

### Conflict of Interest Statement

SR is the author of a Patent Deposit Application regarding the technique used in the functional head impulse test and is a shareholder of a company producing of the fHIT system used in this study (Beon Solutions srl, Zero Branco (TV), Italy). The remaining authors declare that the research was conducted in the absence of any commercial or financial relationships that could be construed as a potential conflict of interest.
